# Studying the Role of Potato Powder on the Physicochemical Properties and Dough Characteristics of Wheat Flour

**DOI:** 10.3390/gels9020073

**Published:** 2023-01-17

**Authors:** Liping Yang, Houfang Zhang, Biao Huang, Shimian Hao, Songnan Li, Peiyan Li, Haibing Yu

**Affiliations:** 1 School of Food Engineering, Anhui Science and Technology University, 9 Donghua Road, Fengyang 233100, China; 2 Joint International Research Laboratory of Agriculture and Agri-Product Safety of the Ministry of Education of China, Institutes of Agricultural Science and Technology Development, Yangzhou University, Yangzhou 225009, China

**Keywords:** potato flour, dough, gluten, rheological properties, fermentation characteristics

## Abstract

Potato flour (PF) is rich in health-promoting compounds that can improve the nutritional benefits of food products after blending with wheat flour. However, the incorporation of PF may influence the processing characteristics of mixed powders and the quality properties of products. In this study, the physicochemical properties, processing characteristics, and structures of mixed powders and their corresponding doughs with different PF content (0%, 10%, 20%, 30%, 40%, 60%, 80%, and 100%) were investigated. The addition of PF dramatically increased the fiber content from 0.09 to 1.10 g·kg^−1^ but diluted the protein in wheat flour. The peak and final viscosity of mixed powders decreased (from 5111.00 to 1806.33 cP and 5195.33 to 2135.33 cP, respectively) with an increase in PF fraction. The incorporation of PF significantly increased gelatinization temperature. The rapidly digestible starch decreased from 30.48% to 19.67%, and resistant starch increased from 16.93% to 41.84% when the PF content increased from 0% to 100%. The water absorption, stability time, and development time decreased with an increase in PF levels. The G′ and G″ of the dough decreased as the addition amount of PF increased, while tan δ presented a complex change tendency. Due to the decrease in protein content in the mixed powders, the addition of PF in wheat flour notably decreased the Hm values of doughs and total carbon dioxide volume produced during fermentation. Additionally, the SH and S–S contents decreased with an increase in PF fraction. Scanning electron microscopy results showed that when the PF content reached up to 80%, a poor and discontinuous gluten framework was formed in the dough. Results showed that PF affected the processing characteristics and gluten structures of wheat dough and was related to the interaction or competition for water molecules between protein and starch, as well as potato starch and wheat starch. Thus, the results of the present study can provide insights into the optimal level of addition of PF during the development of potato-based food products.

## 1. Introduction

Potato (*Solanum tuberosum* L.), the fourth largest crop in world production, has become a major alternative crop in the structural adjustment of agriculture and plays a crucial role in global food security [1]. Potatoes are widely cultivated in China. In 2015, the strategy of promoting potatoes as a staple food was proposed, pushing their application in the production of noodles, steamed bread, and other staple foods. This was aimed at ensuring national food security, increasing agricultural income, improving people’s dietary patterns, and meeting the public’s demand for tasty and healthy food [2]. Compared to other grain crops, potatoes are a low-fat source of nutrients, rich in dietary fiber, and contain balanced amino acids, vitamins, minerals, and antioxidants. Because of their compositional features, potatoes are considered a good substitute for common cereal crops and are gluten-free [2,3]. Thus, using potatoes as raw materials to develop staple food can enrich the nutrition of the products and reduce fat intake in the human body, as well as the higher dietary fiber content can help in overcoming chronic diseases, such as obesity, hypertension, diabetes, cardiovascular and cerebrovascular diseases, and colon cancer.

Potato flour (PF), a fine granular product, usually is obtained by peeling, drying, and grinding and is comprised of all the dry matter in potatoes [4]. As an important form of potato applied in the food industry, PF has been used to develop various products, such as cookies [5], noodles [4,6], bread [2,7], steamed bread [8,9], and other foodstuffs [10]. However, many studies have found that adding a higher amount of PF negatively affects the physical, sensory, and edible qualities of the final product. For instance, Nawaz et al. [6] studied the incorporation effect of PF on the physicochemical and rheological characteristics of dough and the cooking quality and structural properties of instant noodles; results suggested that the addition of 40% PF was preferable. Tao et al. [4] found that the addition of potato powder destroyed the network structure of the gluten and increased the cooking loss of noodles, and pointed out that adding 15–20% potato powder was acceptable. Thus, the drawbacks reported in these studies severely limit the use of PF in food development and restrict the growth of the “potato as a staple food” policy [2]. Furthermore, there are some discrepancies existing in many studies regarding the mechanism of influence of PF on food products. According to most studies, the bad effect of potato powder on product quality is due to the steric intervention effects of PF, which obstruct the formation of the gluten network. However, some researchers propose that the steric intervention effects may not be the major factor contributing to the deterioration of bread quality [2]. Additionally, some researchers attribute it to the competition for water molecules between wheat flour and PF during dough preparation, leading to the inability of some gluten particles to absorb water fully and form a gluten network [4]. Moreover, there are some differences in results concerning the influence of the amount of PF addition on dough and product quality. For example, Nawaz et al. [6] found that with an increase in PF addition, the elasticity modulus (G′) decreased; however, Tao et al. [4] observed the opposite trend. These discrepancies in conclusions may be because of the influence of other additives during the research process or differences in the varieties and preparation methods of PF. Furthermore, in these studies, the highest amount of PF added was about 50%, which could make the analysis less comprehensive. Moreover, there are still many challenges to improving the quality of potato-based foods; therefore, this topic has become a research hotspot. Thus, the mechanism underlying the impact of PF on the physicochemical properties and processing quality of dough is still unclear and needs to be further studied.

Thus, in this study, the amount of PF added was taken as a single variable to understand the mechanism related to the influence of the addition of PF on the physicochemical and structural properties of the mixed powders and their corresponding doughs. The PF/wheat flour mass ratio was considered more comprehensively, and the specific design was as follows 0:100, 10:90, 20:80, 30:70, 40:60, 60:40, 80:20, and 100:0 (potato flour/wheat flour, *w*/*w*). This study can provide a theoretical reference for the production of potato-based foods and also lay a theoretical basis for increasing the amount of PF added to potato staple products.

## 2. Results and Discussion

### 2.1. Proximate Composition Analysis

The basic composition of mixed powders with different ratios of PF and wheat flour is shown in Table 1. The moisture, total protein, and lipid contents in PF 0%, i.e., wheat flour, were markedly higher than those of PF 100%, namely potato flour. Conversely, the ash and crude fiber contents of PF were notably higher than those of wheat flour. The total protein content of PF 0% and PF 100% was 1.24 and 0.68 g·kg^−1^, while the crude fiber content was 0.09 and 1.10 g·kg^−1^, respectively. Furthermore, the starch content of PF 0% and PF 100% was 7.45 and 7.12 g·kg^−1^, respectively. These results could be mainly because of the different properties of the raw materials. Based on Table 1, the moisture, total protein, lipid, and starch contents of mixed powders decreased, while the ash and crude fiber contents presented a significantly increasing tendency with an increase in PF proportion. The results were consistent with the findings of Kumar et al. [11], who claimed that this was mainly because PF had less fat, protein, and starch but higher dietary fiber than wheat flour. Therefore, the addition of PF effectively increased the crude fiber content of mixed powder, but the incorporation of PF had a dilutive effect on the protein and starch contents of wheat flour. The proximate composition of the mixed powder samples showed a non-additive effect, except for the crude fiber.

### 2.2. Pasting Properties of Mixed Powders

**Figure 1:**
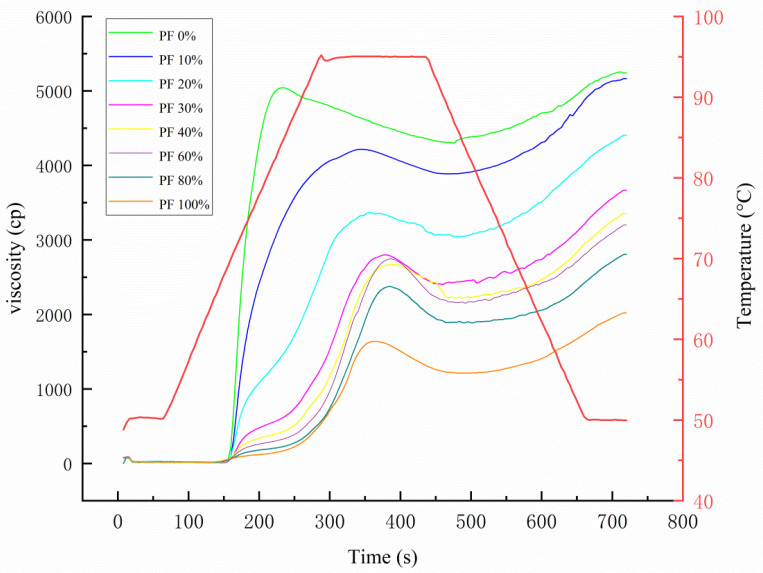
RVA pasting curves of mixed powders with different ratios of potato and wheat flours.

The RVA pasting curves of mixed powders are shown in Figure 1. Based on the pasting curves, the parameters of pasting properties are summarized and presented in Table 2. As seen in Table 2, the pasting properties of mixed powders were significantly influenced by the proportion of PF. The pasting temperatures of mixed powders showed no significant changes, except for PF 80% and PF 100%. The pasting temperatures of PF 80% and PF 100% presented the highest pasting temperatures, up to 75.45 and 76.68 °C, respectively. The increase in pasting temperatures after the incorporation of higher content of PF might be because of the increment in crude fibers and dilution of starch content [12]. The peak and final viscosities of mixed powders decreased with an increase in PF fraction, while the breakdown showed a fluctuating change tendency that was not significant, but the overall trend was downward. The peak viscosity dramatically decreased from 5111.00 cP in PF 0% to 1806.33 cP in PF 100%, and final viscosity significantly decreased from 5195.33 cP in PF 0% to 2135.33 cP in PF 100%, while breakdown decreased from 807.67 cP in PF 0% to 473.33 cP in PF 100%. Cao et al. [13] reported similar findings, i.e., the addition of potato pulp resulted in a decrease in the overall viscosity parameters. This may be attributed to the reduction of the starch concentration in the mixed powders arising from the incorporation of PF as the starch content in PF (7.12 g·kg^−1^) was indeed lower than that of wheat flour (7.45 g·kg^−1^). Furthermore, Liu et al. [14] claimed that the viscosity parameters were positively correlated with starch content. Additionally, Cao et al. [13] reported that dietary fiber could restrain the swelling of starch granules and, accordingly, decrease friction, resulting in low viscosity. In this study, the crude fiber content of mixed powders increased significantly with increasing PF proportion. This also can explain the decrease in viscosity parameters. The setback of mixed powders showed an initial rise, followed by a fall as the PF proportion increased. The lower the setback value, the lower the retrogradation tendency, indicating that less retrogradation of amylose is taking place. The setback value showed a non-additive effect, which might be due to the fact that the factors influencing it, such as leached amylose, fiber, protein, and lipid contents, are complex. This made the setback value to not present a consistent trend of change. Cao et al. [13] found that adding potato pulp lowered the starch content in mixed samples, leading to a decrease in amylose content and a reduction in setback during cooling. Additionally, Xu et al. [15] reported that the setback decreased markedly with increasing wheat gluten content. In general, the addition of PF had a detrimental effect on the pasting behavior of wheat flour.

### 2.3. Thermal Properties of Dough

The gelatinization parameters of mixed powder samples are presented in Table 3. As observed in Table 3, the To, Tp, and Tc significantly increased, especially for the mixed powder samples with higher PF proportion. The PF 100% showed the highest To, Tp, and Tc, which were 61.64, 64.41, and 67.02 °C, respectively. On the one hand, this might be due to the PF being more resistant to swelling and rupturing than wheat flour. Liu et al. [16] found that the pasting temperature exhibited a decreasing tendency with the incorporation of flaxseed flour, which was attributed to the fact that wheat flour was more resistant to swelling and rupturing than flaxseed flour. On the other hand, the increase in gelatinization temperatures of mixed powders might be related to the increase in crude fibers [12]. The gelatinization enthalpy (ΔH) of mixed powders significantly increased with an increase in PF fraction. The ΔH increased from 1.91 J·g^−1^ in PF 0% to 9.88 J·g^−1^ in PF 100%. This might be connected to the gluten protein content of the mixed powder systems because the total protein decreased from 1.24 g·kg^−1^ in PF 0% to 0.68 g·kg^−1^ in PF 100%. This result was in accordance with the report of Xu et al. [15], who observed that the endothermic enthalpy decreased with increasing proportion of gluten.

### 2.4. In Vitro Starch Digestibility

**Figure 2:**
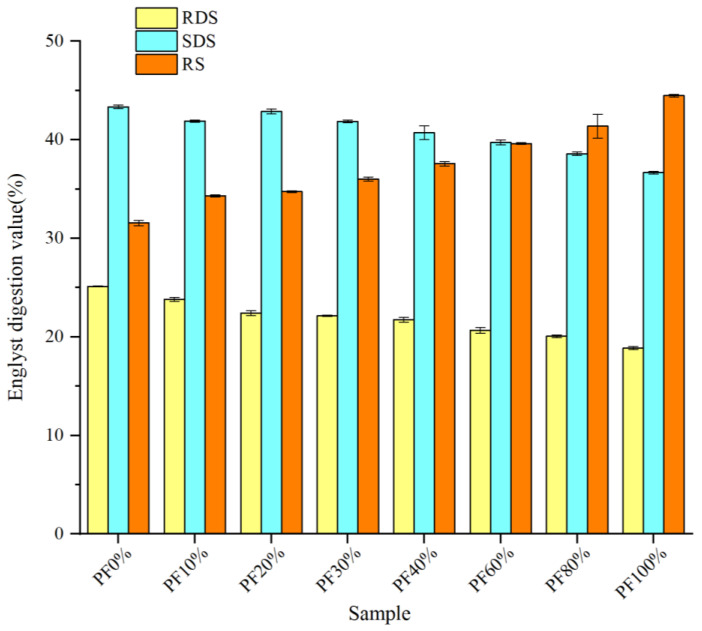
Englyst digestion values of mixed powders with different ratios of potato and wheat flours. RDS, rapidly digestible starch; SDS, slowly digestible starch; RS, resistant starch. Values are expressed as means of three replications. The significant differences between means are at the 95.0% confidence level, and standard deviations are shown in the form of bars.

The RDS, SDS, and RS contents of mixed powders are presented in Figure 2. Compared with PF 0%, the RDS, SDS, and RS contents showed significant changes in mixed powders with the incorporation of PF. The RDS and SDS contents significantly decreased from 25.12% in PF 0% to 18.87% in PF 100% and from 43.34% in PF 0% to 36.66% in PF 100%, while the RS content dramatically increased from 31.54% in PF 0% to 44.47% in PF 100%. These results were similar to those reported by Cao et al. [3], wherein the steamed bread prepared with potato pulp exhibited higher RS and lowered RDS content than normal steamed bread. They proposed that it might be because the non-starch polysaccharides in PF provided non-specific binding sites for α-amylase during the digestion process and, therefore, delayed the rate of starch digestion. Liu et al. [17] also found that the RDS and SDS contents reduced with the incorporation of PF, and they speculated that it was because the starch granules of potatoes were generally larger than those of other plant sources, reducing the specific volume and surface area of starch particles and causing the physical structure to be denser and decreasing the possibility of the enzyme to be in contact with starch. Cappa et al. [18] reported that the larger particle size of starch hindered the access of enzymes deeper into the granule, reducing their contribution to RDS. Additionally, Englyst et al. [19] reported that potato starch contained higher RS content, which helped to induce satiety and produce low glycemic responses in consumers. This can be beneficial for weight control, especially for individuals with type-2 diabetes. Hence, developing foods with PF may confer health benefits.

### 2.5. Dough Mixing Properties

Table 4 presents the mixing and pasting properties of mixed powders. The water absorption (WA) of wheat flour and mixed powders showed significant differences. Increasing PF levels decreased WA, showing values ranging from 60.00% (PF 0%) to 55.20% (PF100%). The WA is influenced by many elements, such as gluten, fiber, and starch. During dough formation, the gliadin and gluten absorb water, swell, and bond to each other to form gluten, while the starch absorbs water, expands, and is wrapped in the gluten network. In addition, the crude fiber absorbs water. Xu et al. [15] reported that the gluten protein possessed a stronger water absorption ability than starch, which explained the changes in the WA of mixed powders. Similar results were observed in Iancu’s [20] study, wherein the addition of potato pulp decreased the WA.

The values of stability time (ST) significantly decreased with an increase in PF levels. The ST represents the strength of dough [13]. Thus, the incorporation of PF negatively influenced dough stability, and Cao et al. [13] speculated that the decreased ST was mainly because of the decrease in protein content due to PF addition. This result was similar to the discovery of Sui et al. [21], who observed that the bran addition resulted in gluten dilution and further contributed to the decrease in dough stability. Thus, the protein content may be the main factor influencing the dough mixing characteristics, especially stability, as the protein content decreased with the incorporation of PF. Based on ST results, PF addition up to 40% was acceptable.

The addition of PF significantly changed the C1 time of dough samples. C1 time represents the development time of dough (i.e., the time interval between the water addition and the time while the dough attains its optimal viscosity and elasticity). As shown in Table 4, the C1 time reduced from 3.87 to 0.61 min with the increase in the PF levels from 0% to 100% in mixed powders. However, PF 80% showed an irregular change, which suddenly increased to 2.61 min. According to Pu et al. [22], the higher the development time, the stronger dough formed, and the strength of the dough was related to the gluten network. Thus, the increase in PF proportion decreased the C1 time, primarily due to the decrease in gluten content in the dough. Additionally, in PF 80%, the change in C1 time may be elucidated by the complex impact of the protein and fiber content as the rate of water absorption and swelling decreased when the fiber content was higher and led to a higher development time of dough. The C3, C3-C4, and C5 represent the pasting characteristics of starch in the dough during the process of heating and cooling cycles [23]. All these parameters increased with the increasing PF content in the dough samples. C3, the maximum torque during the heating phase, reflects the degree of starch gelatinization. The addition of PF to wheat flour led to the increase in C3 value from 1.86 N/m (PF 0%) to 2.89 N/m (PF 100%), which could mainly stem from the lower starch content after the incorporation of PF (presented in Table 1). The C3-C4 value represents the amylase activity or hot-gel stability of dough, and it increased as the PF proportion decreased, indicating that the potato starch gel was more stable. The result was consistent with the breakdown analysis presented in Table 2. The maximum torque during the cooling process (C5) represents the retrogradation of starch paste. The change in the trend of C5 values was similar to the result obtained by Xu et al. [15], who found that C5 increased with an increase in potato starch content of the doughs. Furthermore, the increased C5 values were not good for PF application.

### 2.6. Rheological Properties of Dough

**Figure 3:**
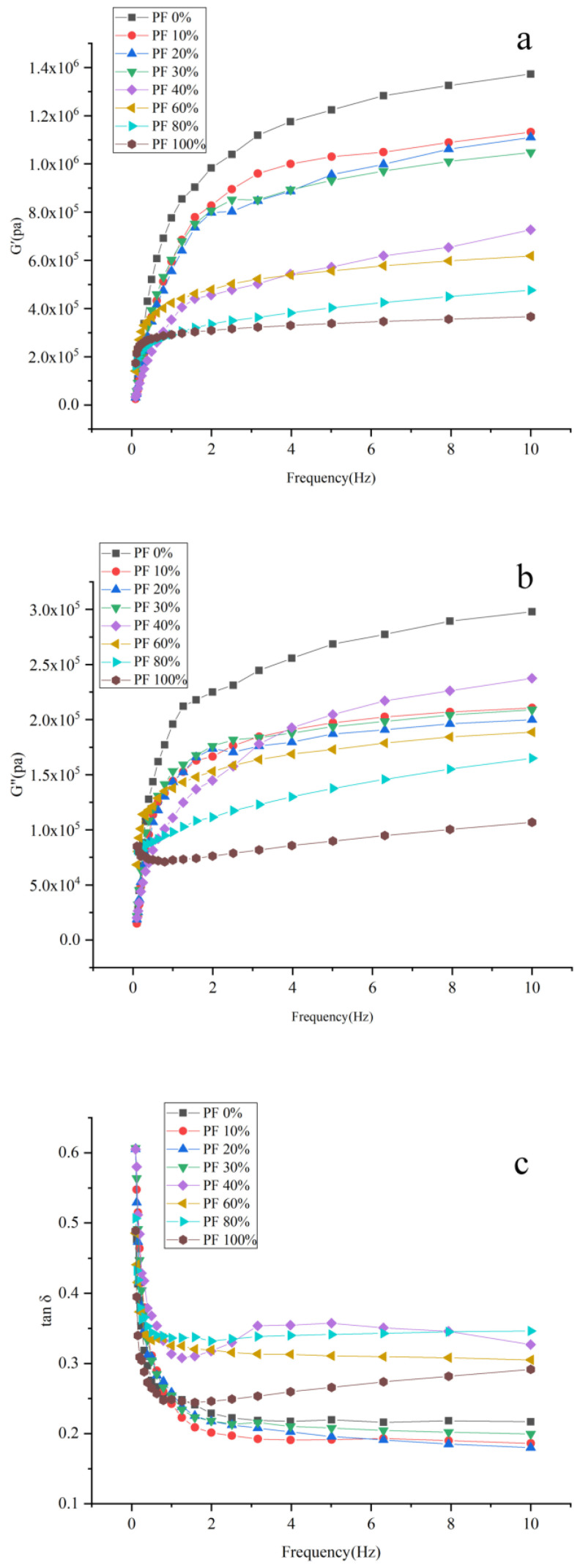
Dynamic shear curves of mixed powders with different ratios of potato and wheat flours at 25 °C. (**a**) G′ vs. frequency plot; (**b**) G″ vs. frequency plot; (**c**) Tan δ vs. frequency plot.

Rheological characteristics have been widely investigated to forecast the processing properties and quality of food products [6]. A frequency sweep test was performed to study the effect of PF on the rheological parameters, i.e., elastic modulus or storage modulus (G′), viscous modulus or loss modulus (G″), and loss tangent (tan δ) of the dough. The G′, G″, and tan δ were plotted against frequency and are shown in Figure 3. Figure 3 showed that the G′ and G″ reduced with an increase in PF addition, suggesting that the incorporation of PF influenced the dough viscoelasticity. This result was consistent with the findings of the study by Tao et al. [24], who determined that the rheological properties of dough mainly depended on its gluten and starch contents. The incorporation of PF diluted the protein in the mixture and decreased the overall gluten content. As competition for water molecules existed between starch and gluten, this further led to not all gluten molecules absorbing enough water to form a gluten network [24]. This could weaken the dough structure and affect the viscosity and elasticity, resulting in a decrease in G′ and G″. However, the change in the trend of G′ and G″ in this study was opposite to the previous findings of Tao et al. [4], who found that the G′ and G″ values increased as the amount of potato powder increased. This might be because Tao et al. [4] used Xuehua PF as the material, in which the starch gelatinized and formed a colloid-like substance, increasing the storage modulus.

The loss factor, tan δ, presented a complex change in its trend. When the proportion of PF added was less than 30%, the tan δ of wheat flour dough was higher than that of mixed powder doughs; however, when the addition amount was more than 40%, the tan δ of mixed powder doughs increased compared to that of wheat flour dough. The variation trend of tan δ was similar to the result of Tao et al. [24], who reported that compared to wheat flour dough, the addition of potato starch (0% to 30%) led to a decrease in tan δ. With the increase in PF addition, tan δ showed a trend of an initial increase, followed by a decrease and then again an increase. This complex trend might be because of the complex influence of the non-starch components in PF, such as lipid, fiber, and protein, on dough viscoelasticity. The tan δ value was consistently less than 1 in the whole frequency range tested, i.e., the Gʹ was higher than the Gʹʹ value. This revealed that the elastic characteristics predominated over viscous characteristics; thus, the material behaved more like a solid [25].

### 2.7. Fermentation Characteristics Analysis of Dough

**Figure 4:**
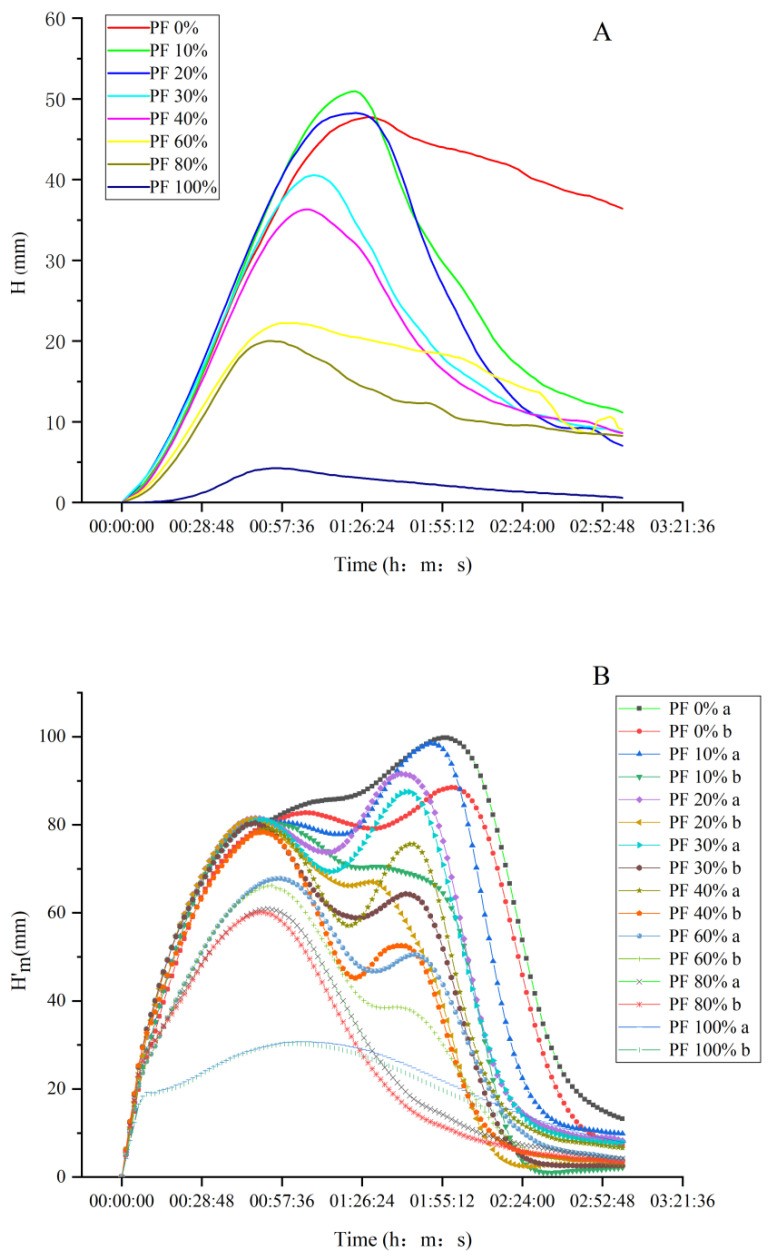
(**A**) Fermentation curves of doughs prepared from mixed powders with different ratios of potato and wheat flours, analyzed using F4 rheofermentometer. (**B**) Gas production and retention in doughs prepared from mixed powders with different ratios of potato and wheat flours, analyzed using F4 rheofermentometer; “a” represents mean gas production and “b” represents mean gas retention.

Dough development and gas behavior measured using a rheofermentometer are shown in Figure 4 and Table 5. The “Hm”, i.e., the maximum height during development time, is an important parameter when assessing the processing characteristics of flour used to develop fermented foods, such as steamed bread or bread [26]. From Table 5, the addition of PF into wheat flours notably decreased the Hm values of doughs, except in PF 10%, which presented a slight increase. This might be because of the decrease in protein content caused by the incorporation of PF. Moreover, the fiber induced by the addition of PF might compete with proteins for water absorption, resulting in water migration from the gluten network to fiber and further leading to inferior gluten network building. The “h” value (dough height at the end of the test) also decreased from 36.40 mm in PF 0% to 0.60 mm in PF 100%, which might be because the PF restricted the dough extension during fermentation [18]. The (Hm–h)/Hm was inversely related to dough stability. As seen in Table 5, the (Hm–h)/Hm dramatically increased from 24.70% in PF 0% to 86.95% in PF 100%, suggesting that the dough stability markedly decreased with the incorporation of PF. This result was in accordance with the Mixolab analysis. Liu et al. [17] reported that this was possibly due to the PF diluting the gluten structure, impeding the dough formation, reducing the viscoelasticity (as shown in Figure 2), and forcing gas to diffuse in all directions.

The maximum height of CO_2_ production (Hm′) and total volume of CO_2_ produced during fermentation (Vt) progressively and significantly decreased with the incorporation of PF. This might be due to the decrease in the amylase content with the incorporation of PF, which reduced its ability to generate fermentable sugars during the process of kneading and fermentation. Accordingly, yeasts might have difficulty growing and reproducing and producing CO_2_ with PF incorporation. This result was confirmed by Liu et al. [17], who pointed out that with an increase in PF concentration, the content of alpha-amylase decreased significantly, affecting the total volume of gas. Xu et al. [26] found that the yeasts had difficulty growing and producing CO_2_ when superfine-ground bran was added compared to bran with bigger particle size, which was attributed to the deactivation of amylase during superfine-grinding. With an increase in PF content, the T_x_, i.e., the time of dough porosity appearance, showed no significant differences, with values ranging from 47.15 to 59.87 min. However, the samples PF 80% and PF 100% showed no detected T_x_ value, revealing that no porosity appeared during fermentation, which could be attributed to gluten deficiency and unstable dough.

### 2.8. Determination of Free SH and S-S

**Figure 5:**
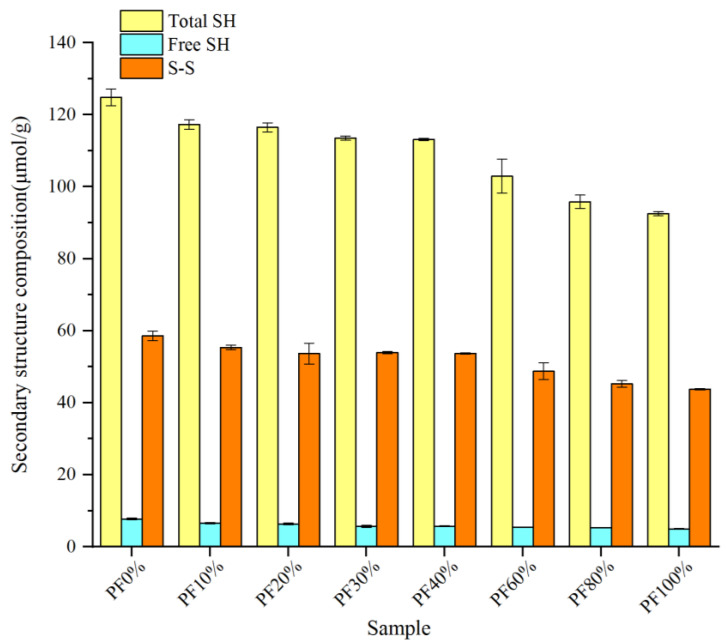
Total sulfhydryl groups (total SH), free sulfhydryl groups (free SH), disulfide bonds (S-S), and secondary structure composition of doughs prepared from mixed powders with different ratios of potato and wheat flours. Values are expressed as means of three replications. The significant differences between means are at the 95.0% confidence level, and standard deviations are shown in the form of bars.

Free SH and S-S greatly affect the structural characteristics and functional properties of gluten [27]. S-S bonds play a decisive role in holding the stability of the dough gluten structure [28]. The influence of PF on free SH and S–S contents in gluten is presented in Figure 5. The content of free SH in the dough gluten was reduced when PF was added. It significantly decreased from 7.72 μmol/g in wheat flour (i.e., PF 0%) to 5.00 μmol/g in PF 100%. This result contradicted the findings of Cao et al. [3], who reported that the free SH content in the doughs increased from 4.83 μmol/g for the wheat flour sample to 6.56 μmol/g when 50% potato pulp was incorporated. The content of S–S showed a similar trend to free SH, decreasing from 58.56 μmol/g in PF 0% to 43.76 μmol/g in PF 100%. This was similar to a previous study conducted by Cao et al. [3], wherein they observed that the S-S content decreased from 19.26 μmol/g to 14.79 μmol/g. Cao et al. [3] pointed out that the unbounded-free SH participated in the formation of S–S; thus, the increased free SH content with increasing PF content mainly originated from the destruction of disulfide bonds. However, in this study, the free SH and S–S contents exhibited a decreasing trend. In theory, the decrease in free SH content suggests less breakage of S-S, i.e., the presence of higher S-S content in the sample. In this study, the decreased S-S content could be mainly ascribed to the reduced levels of gliadin and glutelin, which were diluted with the addition of PF. As the gliadin and glutelin are connected by disulfide bonds to form the gluten network, an increased S–S content corresponds to a more stable gluten structure. Thus, this result indicated that PF incorporation reduced the stability of the dough.

### 2.9. Microstructural Analysis of Dough

**Figure 6:**
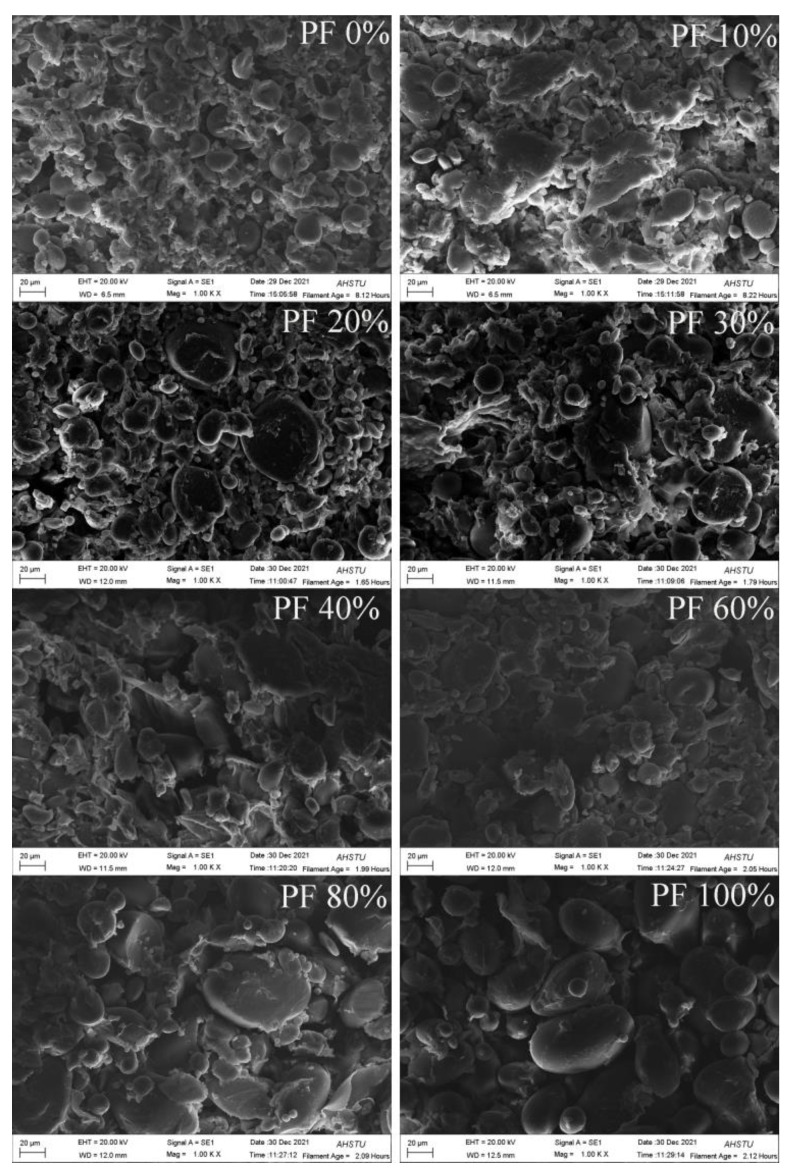
SEM images of doughs prepared from mixed powders with different ratios of potato and wheat flours.

SEM is a better technique to ascertain the interaction of starch and gluten in the dough matrix. Figure 6 shows the SEM images of doughs prepared from mixed powders with varied wheat flour and PF ratios. When the PF content was less than 60%, a relatively intact gluten network could be observed. In particular, for PF 0% to PF 40%, round starch particles were completely encased in the dough gluten matrix, which was typical of the dough structure, suggesting the formation of a good network between starch and protein. When the PF content was up to 20%, larger starch particles were exposed outside the gluten network. These larger granules were potato starch, and because of their big particle size, the gluten might have difficulty wrapping them. When the PF content was higher than 40%, more gaps appeared in the gluten-starch system, resulting in the structure to become loose. When the PF content was up to 80%, the surface connection between starch particles and gluten strands in the dough was reduced, forming a poor and discontinuous gluten skeleton. The starch particles were basically exposed outside the gluten network and were in a dissociated state. The reason might be that the higher the amount of PF added, the weaker the connection between starch and protein [11]. Additionally, the fiber was also destructive to the dough structure. The higher the PF content, the higher the fiber content, and the stronger the damage to the dough network structure. Furthermore, the hygroscopicity of the potato starch might have prevented gluten from absorbing enough water to form a gluten network, negatively affecting the structural stability of the gluten network [24].

## 3. Conclusions

This study proved that PF affected the processing characteristics and gluten structure of wheat flour dough. The addition of PF significantly increased the crude fiber content in mixed powders but decreased their protein and starch contents. The pasting properties of mixed powders dramatically changed after the addition of PF as the peak and final viscosities of mixed powders decreased with an increase in the PF fraction. The addition of PF had a detrimental effect on the pasting behavior of wheat flour, even with 10% addition amount. The gelatinization temperature of mixed powders markedly increased with the addition of PF, which could be mainly attributed to the high fiber content. The starch digestibility of samples presented marked changes: the RDS decreased, while RS increased as the PF content increased from 0% to 100%, and the decrease in starch digestibility was mainly related to the large size of potato starch granules and the blocking effect of fiber in PF. The water absorption and development time of doughs were reduced with an increase in PF levels, which affected its processing characteristics. Furthermore, the G′ and G″ of the dough reduced with an increase in PF content. The incorporation of PF dramatically decreased the Hm value of the dough and the total volume of CO_2_ produced during fermentation. When the PF content was higher than 40%, the gluten-starch system became loose, and when the PF content was up to 80%, a poor and discontinuous gluten framework was formed in the dough. Overall, the weakening effect of PF addition on the product quality was not only due to the decrease in protein content but also related to the fiber content and the interaction between starch and protein. In conclusion, these results might provide a more comprehensive theoretical basis for a better understanding of the influence of PF on the physicochemical, nutritional, and processing properties, as well as the structure of mixed powders and corresponding doughs. In addition, this study can provide information to improve the quality of potato-based products.

## 4. Material and Methods

### 4.1. Materials

High-gluten wheat flour (34% gluten content) was provided by Anhui Fengbao Group Co. Ltd. Potato (*Atlantic*), Hefei, China, suitable for the development of whole PF and potato chips, was procured from the local supermarket. Porcine pancreas α-amylase (P7545, 8 × USP) and amyloglucosidase (A7095, ≥300 U/mL) were purchased from Sigma–Aldrich Co. (St. Louis, MO, USA). All other chemicals used in this study were of analytical grade.

### 4.2. Preparation of PF

The potatoes were thoroughly washed, peeled, and cut into slices. Then the potato slices were soaked in a pre-prepared solution (0.05% sodium bisulfite, 0.4% sodium chloride, and 0.8% citric acid) for 30 min to prevent browning. The potato slices were removed, dried, ground, and then passed through a 100-mesh sieve to obtain the PF.

### 4.3. Flour Blending

The PF and wheat flour were taken in the following mass ratios (*w*/*w*): 0:100, 10:90, 20:80, 30:70, 40:60, 60:40, 80:20, and 100:0, which were labeled as PF 0%, PF 10%, PF 20%, PF 30%, PF 40%, PF 60%, PF 80%, and PF 100%, respectively.

### 4.4. Proximate Composition Analysis

The moisture, fat, total protein, ash, and crude fiber contents of mixed powders were measured following the standard [29] methods. The nitrogen content was measured using an automatic Kieldahl apparatus (Kjeltec™ 8400, Foss Inc., Herisau, Sweden), and the nitrogen conversion factor was set as 6.25.

### 4.5. Pasting Properties of Mixed Flour

The pasting properties of mixed powders were determined using a rapid viscosity analyzer (RVA-TecMaster, Newport Scientific Pty. Ltd., Warriewood, Australia) according to the method of Yang et al. [30] with slight modifications. Three grams of mixed powders were placed into the RVA canister and dispersed in distilled water (25.0 mL). The test procedures were consistent with the description of Yang et al. [31]. The suspensions were equilibrated at 50 °C for 1 min, heated to 95 °C within 4 min, held at 95 °C for 5 min, cooled to 50 °C within 4 min, and held at 50 °C for 1.5 min. The measurements for pasting temperature, peak, setback, breakdown, and final viscosity were then obtained.

### 4.6. Differential Scanning Calorimetry (DSC) Analysis of Mixed Powder

The gelatinization properties of mixed powders were determined using DSC (Model DSC3, Mettler-Toledo Co., Zurich, Switzerland) according to the method described by Yang et al. [30]. The onset, peak, and end gelatinization temperatures and gelatinization enthalpy, i.e., To, Tp, Tc, and ΔH were obtained, respectively.

### 4.7. In Vitro Starch Digestibility

The in vitro starch digestibility of mixed powders was performed following the procedure of Ding et al. [32]. The glucose content was determined using a glucose assay kit. The rapidly digestible starch (RDS), slowly digestible starch (SDS), and resistant starch (RS) were calculated using the following formulas:

(1) RDS (%)=(G20 − G0) × 0.9 × 100

(2) SDS (%(=(G120 − G20) × 0.9 × 100

(3) RS (%)=100 − RDS − SDS

where G20 represents glucose released after 20 min; G120 represents glucose released after 120 min; G0 represents free glucose.

### 4.8. Dough Mixing Properties

The mixing properties of samples were measured using MIXOLAB 2 Mixolab analyzer (France Chopin Technology Co., Ltd., Paris, France) according to the method of Liu et al. [16] and Xu et al. [26] with some modifications. The distilled water was added to the mixed powder to prepare the dough. The dough was subjected to dual-mixing at 80 rpm, and the water tank was maintained at 30 °C. The parameters related to mixing properties were then recorded.

### 4.9. Dough Preparation

Water was added to the mix powder, with the amount of water depending on the water absorption determined by the Mixolab analyzer. The dough was prepared by stirring the flour in a dough mixer (SJJ-D08G1, Little Bear Electric Appliance Co., Ltd., Guangdong, China) for 10 min at 50 rpm. No other substances were mixed to reduce the variables affecting water distribution within the dough.

### 4.10. Rheological Properties of Dough

Dynamic rheological properties of dough were determined using a rheometer (HAAKE RheoStress 6000, Thermo Scientific, Waltham, MA, USA) equipped with 20 mm-diameter parallel plate geometry following the procedure reported by Yang et al. [33].

### 4.11. Determination of Dough Fermentation Characteristics Using Rheofermentometer

The dough fermentation properties were analyzed using rheofermentometer F4 (Chopin Technologies, Villeneuve, France). The dough samples were prepared according to the manipulative instructor of rheofermentometer F4 using an alveograph instrument. Dough (315 g) was placed in the rheofermentometer basket, and a piston (2 kg) was put on the top. The rheological experiments were conducted at 28.5 °C for a period of 3 h. The following parameters were recorded: Hm (mm), the maximum height at development time; h (mm), the height of dough at the ending; Hm′ (mm), the maximum height of CO_2_ production; Vt, the total volume of gas produced during the whole fermentation process.

### 4.12. Determination of Free Sulfhydryl Groups and Disulfide Bonds

Dough samples were freeze-dried and ground to determine the free SH and total SH contents. Fifteen milligrams of the sample were suspended in 1.0 mL of tris-glycine buffer containing 3 mM EDTA, 0.1 M glycine, and 0.1 M Tris-HCl (pH 8.0). Next, 4.7 g of guanidine hydrochloride was added and diluted to 10 mL with the buffer. Finally, the free sulfhydryl groups (-SH) and disulfide bond (S-S) contents in the gluten were determined using the spectrophotometric assay method described by Yang et al. [33].

### 4.13. Microstructure of Dough

The dough was prepared according to the method described in Section 2.9. The scanning electron microscopy (SEM) micrographs of dough samples were obtained using a cold field-emission SEM (EVO-18, Carl Zeiss Germany, Japan), operated at 20.0 kV accelerating voltage, based on the method described by Yang et al. [33].

### 4.14. Statistical Analysis

All analyses were conducted in triplicate and analyzed using SPSS statistical software version 17.0 (SPSS Inc., Chicago, IL, USA). One-way analysis of variance followed by multiple range tests was performed to determine the significant differences between means at 95.0% confidence level.

## Figures and Tables

**Table 1:** Proximate composition of mixed powders with different ratios of potato and wheat flours.

Samples	Moisture (g·kg^−1^)	Ash (g·kg^−1^)	Total Protein (g·kg^−1^)	Lipid (g·kg^−1^)	Crude Fiber (g·kg^−1^)	Starch (g·kg^−1^)
PF 0%	1.05 ± 0.00 ^a^	0.04 ± 0.00 ^h^	1.24 ± 0.01 ^a^	0.13 ± 0.00 ^a^	0.09 ± 0.00 ^h^	7.45 ± 0.01 ^a^
PF 10%	1.05 ± 0.00 ^a^	0.06 ± 0.00 ^g^	1.18 ± 0.06 ^b^	0.11 ± 0.01 ^b^	0.19 ± 0.00 ^g^	7.42 ± 0.03 ^ab^
PF 20%	0.97 ± 0.00 ^b^	0.07 ± 0.00 ^f^	1.17 ± 0.02 ^b^	0.09 ± 0.00 ^c^	0.32 ± 0.00 ^f^	7.38 ± 0.03 ^abc^
PF 30%	0.94 ± 0.00 ^c^	0.09 ± 0.00 ^e^	1.08 ± 0.02 ^c^	0.08 ± 0.00 ^d^	0.45 ± 0.00 ^e^	7.35 ± 0.10 ^bc^
PF 40%	0.91 ± 0.01 ^d^	0.13 ± 0.01 ^d^	1.02 ± 0.01 ^d^	0.07 ± 0.00 ^e^	0.55 ± 0.00 ^d^	7.32 ± 0.02 ^cd^
PF 60%	0.86 ± 0.00 ^e^	0.19 ± 0.00 ^c^	0.89 ± 0.01 ^e^	0.04 ± 0.00 ^f^	0.77 ± 0.00 ^c^	7.25 ± 0.02 ^de^
PF 80%	0.83 ± 0.00 ^f^	0.24 ± 0.01 ^b^	0.75 ± 0.01 ^f^	0.03 ± 0.00 ^g^	0.96 ± 0.00 ^b^	7.19 ± 0.04 ^ef^
PF 100%	0.79 ± 0.00 ^g^	0.28 ± 0.00 ^a^	0.68 ± 0.06 ^g^	0.02 ± 0.00 ^h^	1.10 ± 0.00 ^a^	7.12 ± 0.02 ^g^

^a–h^ Data are means of triplicate analyses with standard deviation. Means in the same column with different lowercase letters were significantly different (*p* < 0.05).

**Table 2:** Pasting properties of mixed powders with different ratios of potato and wheat flours.

Sample	Pasting Temperature (°C)	Peak Viscosity (cP)	Final Viscosity (cP)	Breakdown (cP)	Setback (cP)
PF 0%	68.53 ± 0.45 ^a^	5111.00 ± 199.23 ^a^	5195.33 ± 127.77 ^a^	807.67 ± 108.21 ^a^	892.00 ± 53.08 ^d^
PF 10%	68.83 ± 0.51 ^a^	4201.33 ± 154.10 ^b^	5188.33 ± 45.71 ^a^	292.00 ± 33.15 ^c^	1279.00 ± 116.50 ^ab^
PF 20%	68.50 ± 0.44 ^a^	3361.67 ± 75.84 ^c^	4444.00 ± 88.22 ^b^	404.00 ± 66.43 ^bc^	1486.33 ± 107.86 ^a^
PF 30%	69.03 ± 0.03 ^a^	2803.00 ± 79.62 ^cd^	3706.00 ± 79.98 ^c^	467.00 ± 63.41 ^bc^	1370.00 ± 93.67 ^ab^
PF 40%	69.03 ± 0.03 ^a^	2672.67 ± 177.02 ^d^	3402.00 ± 185.71 ^cd^	468.00 ± 68.79 ^bc^	1197.33 ± 89.72 ^bc^
PF 60%	68.50 ± 0.43 ^a^	2668.67 ± 181.16 ^d^	3202.00 ± 178.50 ^cd^	617.33 ± 61.42 ^b^	1150.67 ± 110.94 ^bc^
PF 80%	75.45 ± 11.84 ^b^	2536.33 ± 645.61 ^d^	2967.33 ± 644.71 ^d^	578.67 ± 237.55 ^b^	1009.67 ± 237.00 ^cd^
PF 100%	76.68 ± 15.40 ^b^	1806.33 ± 392.72 ^e^	2135.33 ± 300.11 ^e^	473.33 ± 147.08 ^bc^	802.33 ± 59.70 ^d^

^a–e^ Data are means of triplicate analyses with standard deviation. Means in the same column with different lowercase letters were significantly different (*p* < 0.05).

**Table 3:** Thermal properties of mixed powders with different ratios of potato and wheat flours.

Sample	To (°C)	Tp (°C)	Tc (°C)	ΔH (J·g^−1^)	Tc− To (°C)
PF 0%	56.56 ± 0.41 ^c^	60.45± 0.16 ^d^	63.77 ± 0.66 ^b^	1.91 ± 0.78 ^b^	7.21 ± 1.07 ^ab^
PF 10%	58.15 ± 0.06 ^b^	62.58 ± 0.47 ^c^	66.62 ± 0.10 ^a^	2.56 ± 0.38 ^b^	8.47 ± 0.04 ^a^
PF 20%	58.62 ± 0.19 ^b^	63.57 ± 0.13 ^b^	66.62 ± 0.03 ^a^	3.37 ± 0.46 ^b^	8.01 ± 0.16 ^a^
PF 30%	61.41 ± 0.84 ^a^	64.64 ± 0.19 ^a^	66.76 ± 0.40 ^a^	3.59 ± 0.19 ^b^	5.35 ± 1.22 ^c^
PF 40%	61.34± 0.73 ^a^	64.96 ± 0.77 ^a^	67.78 ± 0.64 ^a^	4.69 ± 0.23 ^b^	6.44 ± 0.10 ^bc^
PF 60%	61.55 ± 0.12 ^a^	64.40 ± 0.33 ^a^	66.92 ± 1.21 ^a^	7.83 ± 0.31 ^a^	5.37 ± 1.08 ^c^
PF 80%	61.59 ± 0.47 ^a^	64.33 ± 0.18 ^ab^	66.95 ± 0.57 ^a^	8.00 ± 1.94 ^a^	5.36 ± 0.31 ^c^
PF 100%	61.64 ± 0.27 ^a^	64.41 ± 0.46 ^a^	67.02 ± 0.08 ^a^	9.88 ± 3.77 ^a^	5.39 ± 0.29 ^c^

^a–d^ Data are means of triplicate analyses with standard deviation. Means in the same column with different lowercase letters were significantly different (*p* < 0.05).

**Table 4:** Mixolab parameters of mixed powders with different ratios of potato and wheat flours.

Samples	WA (%) ^1^	ST (min) ^2^	C1 (min)	C3 (N/m)	C3–C4 (N/m)	C5 (N/m)
PF 0%	60.00 ± 1.00 ^a^	7.20 ± 0.10 ^a^	3.87 ± 0.35 ^a^	1.86 ± 0.02 ^g^	0.09 ± 0.04 ^g^	3.46 ± 0.05 ^f^
PF 10%	59.70 ± 0.10 ^a^	5.53 ± 0.15 ^bc^	2.84 ± 0.11 ^b^	2.53 ± 0.04 ^f^	0.21 ± 0.07 ^f^	3.69 ± 0.09 ^d^
PF 20%	58.07 ± 0.15 ^b b^	5.37 ± 0.06 ^c^	1.93 ± 0.02 ^c^	2.64 ± 0.01 ^e^	0.30 ± 0.03 ^e^	3.59 ± 0.01 ^e^
PF 30%	56.30 ± 0.10 ^c^	5.90 ± 0.61 ^b^	1.05 ± 0.04 ^c^	2.90 ± 0.01 ^d^	0.51 ± 0.01 ^d^	3.64 ± 0.01 ^cd^
PF 40%	54.20 ± 0.10 ^d^	3.00 ± 0.10 ^d^	1.03 ± 0.02 ^c^	3.09 ± 0.01 ^c^	0.69 ± 0.03 ^c^	3.62 ± 0.01 ^cd^
PF 60%	53.20 ± 0.10 ^e^	2.23 ± 0.15 ^e^	0.99 ± 0.03 ^c^	3.43 ± 0.01 ^b^	1.00 ± 0.01 ^a^	4.16 ± 0.01 ^c^
PF 80%	49.30 ± 0.10 ^f^	1.74 ± 0.06 ^f^	2.61 ± 0.17 ^b^	4.23 ± 0.01 ^a^	0.93 ± 0.00 ^b^	4.44 ± 0.01 ^b^
PF 100%	55.20 ± 0.10 ^g^	1.25 ± 0.01 ^g^	0.61 ± 0.02 ^d^	2.89 ± 0.01 ^d^	0.17 ± 0.02 ^f^	4.65 ± 0.01 ^a^

^a–g^ Data are means of triplicate analyses with standard deviation. Means in the same column with different lowercase letters were significantly different (*p* < 0.05). ^1^ WA: water absorption. ^2^ ST: stability time.

**Table 5:** Fermentation rheological properties of mixed powders with different ratios of potato and wheat flours.

Dough Samples	Dough Development ^(1)^			Gas Behavior ^(2)^		
	H_m_ (mm)	h (mm)	(H_m_ − h)/H_m_(%)	H_m_′ (mm)	V_t_ (mL)	T_x_ (min)
PF 0%	48.40 ± 1.00 ^b^	36.40 ± 0.60 ^a^	24.70 ± 2.80 ^c^	100.20 ± 2.30 ^a^	1827.50 ± 29.50 ^a^	52.43 ± 10.50 ^ab^
PF 10%	50.95 ± 0.15 ^a^	11.15 ± 1.85 ^b^	78.05 ± 3.55 ^a^	98.50 ± 1.80 ^a^	1637.00 ± 1.00 ^b^	59.87 ± 4.50 ^a^
PF 20%	48.90 ± 2.30 ^ab^	7.05 ± 1.25 ^c^	85.65 ± 1.95 ^a^	91.55 ± 0.65 ^b^	1502.00 ± 5.00 ^c^	47.15 ± 2.15 ^b^
PF 30%	40.75 ± 1.55 ^c^	8.65 ± 1.55b ^c^	78.85 ± 3.05 ^a^	88.30 ± 0.40 ^b^	1448.00 ± 11.00 ^d^	47.87 ± 1.50 ^ab^
PF 40%	36.85 ± 0.35 ^d^	8.60 ± 0.20b ^c^	76.65 ± 0.65 ^a^	79.40 ± 2.30 ^c^	1290.50 ± 2.50 ^e^	50.87 ± 1.50 ^ab^
PF 60%	23.23 ± 0.08 ^e^	9.08 ± 2.53b ^c^	56.70 ± 6.40 ^b^	67.80 ± 0.40 ^d^	1042.50 ± 13.50 ^f^	47.87 ± 7.50 ^ab^
PF 80%	20.15 ± 1.35 ^f^	8.25 ± 0.35 ^c^	58.60 ± 4.60 ^b^	61.35 ± 2.55 ^e^	784.50 ± 26.50 ^g^	/
PF 100%	4.25 ± 0.55 ^g^	0.60 ± 0.60 ^d^	86.95 ± 12.15 ^a^	30.85 ± 0.35 ^f^	596.00 ± 27.00 ^h^	/

(1) Hm (mm), the maximum height at development time; h (mm), dough height at the termination of the test; (H_m_ − h)/H_m_ that is inversely related to dough stability. (2) H_m_′ (mm), the maximum height of CO_2_ production; V_t_, the total volume of CO_2_ produced during fermentation; T_x_ (min), the time of dough porosity appearance.“/” represents undetectable. ^a–h^ Data are means of triplicate analyses with standard deviation. Means in the same column with different lowercase letters were significantly different (*p* < 0.05).

## Data Availability

All basic data supporting the results of this study are available from the corresponding author.
